# Impaired heart rate variability in patients with arrhythmogenic cardiomyopathy: A multicenter retrospective study in China

**DOI:** 10.3389/fcvm.2022.1044797

**Published:** 2022-10-31

**Authors:** Baowei Zhang, Chunjiang Zhou, Jinqiu Liu, Jinlin Zhang, Chenyang Jiang, Min Tang, Jiaxi Xie, Yizhang Wu, Xin Xie, Xiaorong Li, Jinbo Yu, Xuecheng Wang, Dian Cheng, Jian Zhou, Zijun Chen, Fenghua Fan, Xiujuan Zhou, Aibin Tao, Bing Yang

**Affiliations:** ^1^Center of Cardiology, Shanghai East Hospital, Tongji University School of Medicine, Shanghai, China; ^2^Center of Cardiology, Shanghai East Hospital, Nanjing Medical University, Shanghai, China; ^3^Department of Cardiology, The First Affiliated Hospital of Dalian Medical University, Dalian, China; ^4^Department of Cardiology, Wuhan Asia Heart Hospital, Wuhan, China; ^5^Department of Cardiology, Sir Run Run Shaw Hospital, School of Medicine, Zhejiang University, Hangzhou, China; ^6^Department of Cardiology, State Key Laboratory of Cardiovascular Disease, Cardiovascular Institute, Fuwai Hospital, National Center for Cardiovascular Diseases, Chinese Academy of Medical Sciences and Peking Union Medical College, Beijing, China; ^7^Department of Cardiology, The First Affiliated Hospital of Nanjing Medical University, Nanjing, China; ^8^Department of Cardiology, The Affiliated People’s Hospital of Jiangsu University, Zhenjiang, China

**Keywords:** arrhythmogenic cardiomyopathy (ACM), heart rate variability, sympathetic nerve system (SNS), ventricular tachycardia (VT), SDNN

## Abstract

**Background:**

Cardiac sympathetic nerve system (SNS) might play an important role in arrhythmogenesis of arrhythmogenic cardiomyopathy (ACM). This study aims to assess the activity of cardiac SNS in ACM patients by heart rate variability (HRV), and to investigate its predictive value for sustained ventricular tachycardia (sVT).

**Methods:**

A total of 88 ACM patients and 65 sex- and age- matched healthy participants were enrolled. The time domain measures were used to evaluate the activity of cardiac SNS. An independent cohort with 48 ACM patients was as the validation cohort.

**Results:**

ACM patients had lower levels of standard deviation of all NN intervals (SDNN) [118.0 (90.3, 136.8) vs. 152.0 (132.5, 174.5) ms, *p* < 0.001] compared with healthy participants. Further analysis showed ACM patients with sVT had lower levels of SDNN than those without sVT (105.0 ± 28.1 vs. 131.8 ± 33.1 ms, *p* < 0.001). Multivariate logistic regression analysis showed SDNN was independently associated with sVT in ACM patients [odds ratio (OR) 0.59, 95% confidence interval (CI) (0.45–0.78), *p* < 0.001]. Receiver operating characteristics curve demonstrated SDNN had clinical values in predicting sVT in ACM patients [area under the curve (AUC) = 0.73, 95% CI (0.63–0.84), *p* < 0.001], which was verified in the validation cohort.

**Conclusion:**

The present study suggests that HRV is impaired in patients with ACM, and the SDNN level has a moderate value in risk stratification for sVT in ACM patients. In addition, the finding might provide new target for the further management of ACM with integrated traditional Chinese and western medicine.

## Introduction

Arrhythmogenic cardiomyopathy (ACM) is an inherited cardiomyopathy characterized by progressive cardiomyocyte loss and subsequent replacement with fibrofatty tissue in both ventricles, which predispose patients to high risks of ventricular arrhythmias (VAs), sudden cardiac death (SCD), and heart failure (1). The estimated prevalence of ACM is ranged between 1/5,000 and 1/2,000 among different study populations, with a slight male predominant. It is also one of the most common causes of sudden death in young people and athletes (2). For patients with ACM have risks of VAs and SCD, risk stratification aimed to identify patients who have greater likelihood of VAs is of great importance in management of patients with ACM (3).

The cardiac sympathetic nerve system (SNS) plays important roles in the pathogenesis of ACM and the modulation of VAs (4, 5). High incidence of VAs with isoproterenol infusion and benefits of β-blocker treatment on VAs burdens in patients with ACM indicated that the cardiac SNS played key role in the triggering of VAs (6–8). Previous studies employed ^123^I labeled metaiodobenzylguanidine (^123^I-MIBGI) myocardial SPECT showed ACM patients had abnormalities of cardiac sympathetic nervous innervation, which were associated with higher risk of VAs (9, 10). However, this method is not used as a routine tool to assess the cardiac SNS in patients with ACM for its complex procedure and radioactivity. Heart rate variability (HRV), calculated based on the oscillation in the intervals between consecutive heart beats, is the most common method used for indirect assessment of cardiac SNS activity, and has been used to risk stratification in many other cardiovascular diseases routinely (11). However, it is unclear whether HRV has clinical values in patients with ACM. The purpose of this study aimed to assess the HRV in patients with ACM, and investigate its clinical value in risk stratification for VAs in this population.

## Patients and methods

### Patients

A retrospective study was conducted on 129 patients diagnosed with ACM in the department of Cardiology, the First Affiliated Hospital of Nanjing Medical University from January 2006 to February 2015. The study was approved by the institutional ethics committee board of the First Affiliated Hospital of Nanjing Medical University (NO.: 2011-SR-014) and individual consent for this retrospective analysis was waived. In addition, 69 ACM patients from other 4 arrhythmia centers were used as the validation cohort. The diagnosis of ACM was based on the International Task Force Criteria of ACM (12, 13). Sustained ventricular tachycardia (sVT) was defined as recorded spontaneous persistent ventricular tachycardia (lasting for ≥ 30 s at ≥ 100 bpm, or with unstable hemodynamics requiring cardioversion), ventricular fibrillation/flutter, or appropriate ICD intervention. In addition, 65 sex- and age- matched healthy participants were enrolled as the control group. Exclusion criteria included: (1) patients with incomplete data; (2) patients with left ventricular ejection fraction (LVEF) < 35% or other comorbidities with high risk of VAs; (3) diabetics with long-term poor glycemic control or evidence of diabetic neuropathy; (4) patients with chronic obstructive pulmonary disease, severe renal dysfunction, liver dysfunction, thyroid disorder, or malignancy; (5) patients with nervous system disorders (Supplementary Figure 1).

### Data collection and heart rate variability analysis

For all participants, the data about baseline characteristics, which included sex, age, and comorbidities were collected. For patients with ACM, more detailed baseline characteristics, including age at diagnosis, symptoms, anti-arrhythmic drugs (AADs), and other non-pharmacological therapies were collected. Surface electrocardiography (ECG), 24-h Holter recording, and echocardiography were scrutinized by two independent doctors to determine the potential predictors for risk stratification in ACM.

HRV was assessed based on the 24-h Holter recording using a validated three-channel device (Seer Light Dynamic Electrocardiogram record system, GE Healthcare, USA). During the 24-h recording period, all participants were required to maintain their normal activities. All parameters related to HRV were analyzed and calculated on the basis of removal of ectopic beats. The time domain measures were used to evaluate the HRV in this study, which were calculated based on statistical and mathematical analysis on RR intervals. The commonly used variables include the standard deviation of all normal-normal (NN) intervals (SDNN), the standard deviation of the average NN intervals (SDANN), the square root of the mean of the squares of the successive differences between adjacent NN intervals (rMSSD) and the percentage of NN intervals that differ by > 50 ms (pNN50). NN intervals refer to all intervals between adjacent QRS complexes resulting from sinus node depolarization (Supplementary Figure 2). SDNN is the standard deviation of all NN intervals over a 24-h period. SDANN is the standard deviation of the average NN intervals calculated over a 5-min period. Both SDNN and SDANN reflect greater sympathetic nervous contribution to HRV, and decreased levels of SDNN and SDANN indicate increased sympathetic activity. rMSSD is the square root of the mean squared difference of successive NN intervals during a 24-h recording. NN50 is the number of interval differences of successive NN intervals more than 50 ms, and pNN50 represents the percentage of NN50 in the total number of NN intervals. rMSSD and pNN50 are two reliable indicators for parasympathetic activity (14, 15).

### Statistical analysis

Kolmogorov-Smirnov test was firstly employed to determine the distribution patterns of the continuous variables. Continuous variables with normal distribution were presented as mean ± SD and compared with the Student’s *t*-test to assess the differences. Otherwise, median (25th, 75th) was used to present the data, and the Mann-Whitney test was employed to calculate the differences. All the categorical variables were summarized as percentages, and the χ^2^ or Fisher exact test was used to evaluate the differences when appropriate. Univariate and multivariate binary logistic regression analyses were performed to determine the predictors of sVT in patients with ACM. All variables were used in the univariate analyses, and the variables with a significance of < 0.10 were entered into multivariate logistic regression analysis. An automatic stepwise selection procedure using the maximum partial likelihood ratio χ^2^ statistic to enter (*p* ≤ 0.05 level) or remove (*p* > 0.05 level) a covariate into the model was used. Furthermore, receiver operating characteristics (ROC) curve was performed in ACM patients to investigate the value of HRV variables in differentiating patients with sVT from patients without sVT. A two-tailed *p*-value of < 0.05 was taken as significance. All the statistical analyses were performed by the SPSS Statistics for Windows, v26.0 (SPSS Inc., USA) and GraphPad Prism 8.0.2 (GraphPad, USA).

## Results

### Baseline characteristics

There were 88 patients with ACM in the model group were enrolled after 41 patients were excluded for incomplete data (*n* = 14), LVEF < 35% (*n* = 5), with other arrhythmias (*n* = 8) or with other comorbidities which might have effects on HRV (*n* = 14) (Supplementary Figure 1). Patients in the ACM group had comparable age (41.8 ± 12.9 vs. 42.0 ± 15.1 years old) and proportion of male (76.1% vs. 64.6%) to those in the healthy control (HC) group. In addition, there were similar comorbidities between ACM and HC groups (Table 1). More detailed baseline characteristics of patients with ACM were shown in Table 2. Patients in the ACM group were diagnosed with ACM at the mean age of 38.4 years old, and had a mean history duration of 4.5 years. There were 54 patients (61.4%) with right ventricular enlargement, 39 patients (44.3%) with right ventricular outflow tract dyskinesia, and 60 patients (68.2%) with right ventricular free wall dyskinesia. Most ACM patients (94.3%) had normal left ventricular geometry and systolic function (LVEF ≥ 50%). T wave inversion (TWI) in precordial leads (≥3 leads, 51 patients, 58%), inferior leads (≥2 leads, 27 patients, 30.7%), and RBBB (24 patients, 27.3%) on surface ECG were common in ACM patients (Table 2).

**TABLE 1:** Baseline characteristics of participants.

Variables	Healthy control group (*n* = 65)	ACM group (*n* = 88)	*p*-value
Age (years)	42.0 ± 15.1	41.8 ± 12.9	0.93
Male (*n*, %)	42 (64.6)	67 (76.1)	0.15
Hypertension (*n*, %)	12 (18.5)	11 (12.5)	0.36
Diabetes (*n*, %)	2 (3.1)	3 (3.4)	1.0
Coronary artery disease (*n*, %)	0 (0)	2 (2.3)	0.51
Heart failure (*n*, %)	4 (4.5)	0 (0)	0.14
Smoking (*n*, %)	14 (21.5)	17 (19.3)	0.84
Alcohol (*n*, %)	6 (9.2)	10 (11.6)	0.79

ACM, arrhythmogenic cardiomyopathy.

**TABLE 2:** Clinical characteristics of patients with ACM.

Variables	Overall (*n* = 88)	Sustained VT (*n* = 52)	No sustained VT (*n* = 36)	*p*-value
Age (years)	41.8 ± 12.9	42.4 ± 12.4	40.9 ± 12.6	0.60
Male (*n*, %)	67 (76.1)	48 (92.3)	19 (52.8)	<0.001
History (years)	2.0 (1.0, 6.8)	2.0 (1.0, 6.0)	2.0 (1.0, 7.0)	0.78
Age of diagnosis (years)	40.0 (29.3, 47.8)	42.0 (29.3, 46.8)	38.5 (27.0, 48.0)	0.72
Syncope (*n*, %)	34 (38.6)	22 (42.3)	12 (33.3)	0.40
Family history (*n*, %)	10 (11.4)	8 (15.4)	2 (5.6)	0.19
ICD (*n*, %)	8 (9.1)	6 (11.5)	2 (5.6)	0.46
RFCA (*n*, %)	16 (18.2)	12 (23.1)	4 (11.1)	0.15
Hypertension (*n*, %)	11 (12.5)	5 (9.6)	6 (16.7)	0.51
Diabetes (*n*, %)	3 (3.4)	0 (0)	3 (8.3)	0.07
CAD (*n*, %)	2 (2.3)	2 (3.8)	0 (0)	0.51
Heart failure (*n*, %)	4 (4.5)	1 (1.9)	3 (8.3)	0.30
Smoking (*n*, %)	17 (19.3)	15 (28.8)	2 (5.6)	0.007
Alcohol (*n*, %)	10 (11.4)	8 (15.4)	2 (5.6)	0.19
**Treatment with AADs**				
Class I AADs (*n*, %)	17 (19.3)	13 (25)	4 (11.1)	0.11
Class AADs (*n*, %)	18 (20.5)	12 (23.1)	6 (16.7)	0.46
Class AADs (*n*, %)	20 (22.7)	16 (30.8)	4 (11.1)	0.03
Class AADs (*n*, %)	2 (2.3)	2 (3.8)	0 (0)	0.51
**Surface electrocardiography**				
TWI-P (*n*, %)	51 (58.0)	37 (71.2)	14 (38.9)	0.003
TWI-I (*n*, %)	27 (30.7)	22 (42.3)	5 (13.9)	0.004
RBBB (*n*, %)	24 (27.3)	16 (30.8)	8 (22.2)	0.38
Epsilon waves (*n*, %)	15 (17.0)	12 (23.1)	3 (8.3)	0.07
**Measurements on echocardiography**				
LVDD (mm)	48.4 ± 5.1	47.9 ± 5.1	49.1 ± 5.1	0.27
LVEF (%)	65.2 (62.0, 67.5)	64.1 (61.3, 68.0)	65.4 (63.3, 67.3)	0.45
RV enlargement (*n*, %)	54 (61.4)	41 (78.8)	13 (36.1)	<0.001
ROVT dyskinesia (*n*, %)	39 (44.3)	19 (36.5)	20 (55.6)	0.08
RV free wall dyskinesia (*n*, %)	60 (68.2)	37 (71.2)	23 (63.9)	0.47
PAH (*n*, %)	33 (37.5)	24 (46.2)	9 (25)	0.04
TR (*n*, %)	53 (60.2)	35 (67.3)	18 (50)	0.10
MR (*n*, %)	27 (30.7)	14 (26.9)	13 (36.1)	0.36
**Measurements on Holter**				
PVCs (counts/24 h)	1,381 (259, 9,259)	655 (94, 4,011)	4,410 (1,200, 15,383)	0.004
Mean HR (bpm)	68.6 ± 8.8	68.6 ± 9.7	68.7 ± 7.5	0.96
HRmax (bpm)	109 (98, 127)	106 (93, 126)	120 (105, 129)	0.03
HRmin (bpm)	48 (45, 54)	49 (45, 56)	48 (45, 52)	0.30
NSVT (*n*, %)	41 (46.6)	24 (46.2)	17 (47.2)	0.92

AAD, anti-arrhythmic drug; ACM, arrhythmogenic cardiomyopathy; bpm, beats per minute; CAD, coronary artery disease; HR, heart rate; ICD, implanted cardiac defibrillator; LVDD, left ventricular end diastolic dimension; LVEF, left ventricular ejection fraction; MR, mitral regurgitation; NSVT, non-sustained ventricular tachycardia; PAH, pulmonary artery hypertension; PVC, premature ventricular complex; RBBB, right bundle branch block; RFCA, radiofrequency catheter ablation; RV, right ventricular; RVOT, right ventricular outflow tract; TR, tricuspid regurgitation; TWI-I, T wave inversion in inferior leads; TWI-P, T wave inversion in precordial leads; VT, ventricular tachycardia.

### Patients with arrhythmogenic cardiomyopathy had impaired heart rate variability

**FIGURE 1:**
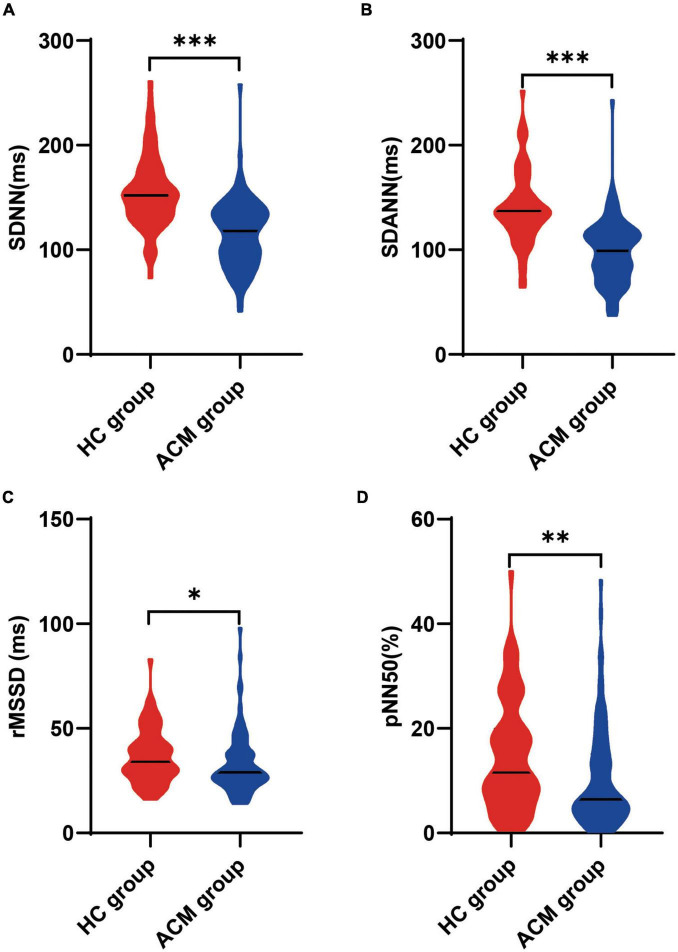
Comparison of heart rate variability between ACM patients and healthy control participants. The Mann-Whitney test was used for assess the difference **(A–D)**, *p < 0.05; **p < 0.01; ***p < 0.001. ACM, arrhythmogenic cardiomyopathy; HC, healthy control; pNN50, percentage of normal RR intervals that differ by > 50 ms; rMSSD, square root of the mean of the squares of the successive differences between adjacent NN intervals; SDANN, standard deviation of the average NN intervals; SDNN, standard deviation of all NN intervals; sVT, sustained ventricular tachycardia.

As variables reflected greater sympathetic nervous contribution to HRV, the levels of SDNN and SDANN were significantly lower in ACM group compared with those in HC group [118.0 (90.3, 136.8) vs. 152.0 (132.5, 174.5) ms, *p* < 0.001 for SDNN; 99.0 (77.0, 117.0) vs. 137.0 (119.5, 153.0) ms, *p* < 0.001 for SDANN, Figures 1A,B], as well as the levels of rMSSD [29.0 (24.0, 38.0) vs. 34.0 (27.5, 44.0) ms, *p* = 0.02] and pNN50 [6.4 (4.0, 15.1) vs. 11.6 (6.9, 19.9)%, *p* = 0.004), (Figures 1C,D]. All these results indicated that patients with ACM had impaired HRV, with increased sympathetic activity combined with decreased parasympathetic activity.

### Decreased levels of standard deviation of all normal-normal intervals in arrhythmogenic cardiomyopathy patients with sustained ventricular tachycardia

Fifty-two patients (59.1%) had recordings of sVT. Compared with patients without sVT, patients with sVT were more male (92.3% vs. 52.8%, *p* < 0.001), higher proportion of smoking (28.8% vs. 5.6%, *p* = 0.007), more common TWI in precordial leads (71.2% vs. 38.9%, *p* = 0.003) and inferior leads (42.3% vs. 13.9%, *p* = 0.004) on surface ECG, higher proportion of RV enlargement (78.8% vs. 36.1%, *p* < 0.001) and PAH (46.2% vs. 25%, *p* = 0.04) on echocardiography (Table 2).

**FIGURE 2:**
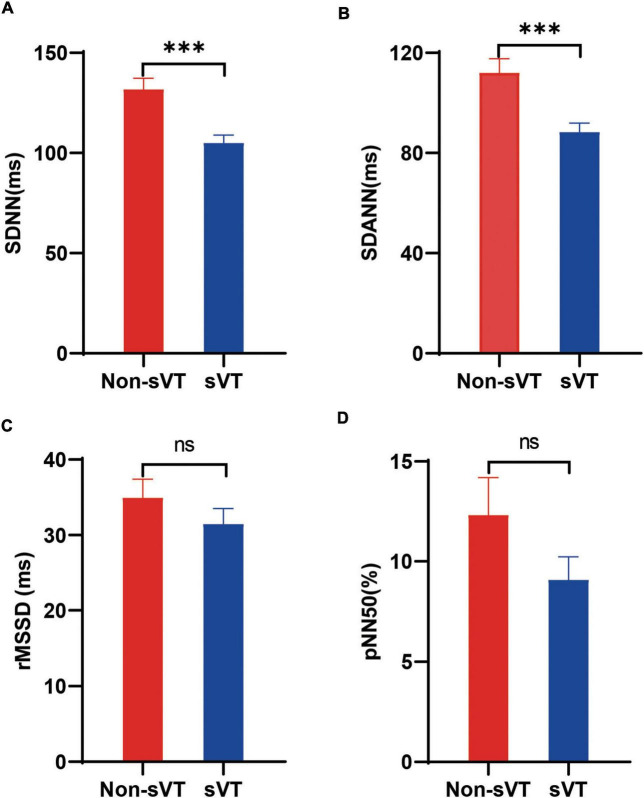
Comparison of heart rate variability between ACM patients with and without sVT. The Student’s *t*-test **(A,B)** and Mann-Whitney test **(C,D)** was used for assess the difference. ***p < 0.001. Bars in the figures indicate standard error. ACM, arrhythmogenic cardiomyopathy; pNN50, percentage of normal RR intervals that differ by > 50 ms; rMSSD, square root of the mean of the squares of the successive differences between adjacent NN intervals; SDANN, standard deviation of the average NN intervals; SDNN, standard deviation of all NN intervals; sVT, sustained ventricular tachycardia; ns, not significant.

Comparing the HRV variables between sVT group and non-sVT group showed that the levels of SDNN and SDANN in patients with sVT were lower significantly than those in patients without sVT (105.0 ± 28.1 vs. 131.8 ± 33.1 ms, *p* < 0.001 for SDNN; 88.4 ± 25.7 vs. 112.1 ± 33.6 ms, *p* < 0.001 for SDANN; Figures 2A,B). However, the difference of neither rMSSD nor pNN50 was significant between the two groups (Figures 2C,D).

### Standard deviation of all normal-normal intervals was independently associated with sustained ventricular tachycardia in arrhythmogenic cardiomyopathy patients

Univariate logistic regression analysis showed that male, history of smoking, TWI in precordial leads and inferior leads on surface ECG, RV enlargement, PAH on echocardiography, PVC counts on 24-h Holter, SDNN, and SDANN were associated with sVT in ACM patients (Table 3). However, only SDNN [SDNN/10, OR 0.59, 95% CI (0.45–0.78), *p* < 0.001], male [OR 17.72, 95% CI (3.49–89.90), *p* = 0.001], TWI in precordial leads [OR 3.76, 95% CI (1.04–13.58), *p* = 0.04] and inferior leads [OR 4.77, 95% CI (1.07–21.18), *p* = 0.04] on surface ECG were independently associated with sVT in ACM patients by multivariate logistic regression analysis (Table 3).

**TABLE 3:** Univariate and multivariate logistic regression analyses of predictors of sustained VT in patients with ACM.

Variables	Odds ratio	95% CI	*p*-value
**Univariate analysis**			
TWI-P	3.88	1.58–9.53	0.003
TWI-I	4.55	1.52–13.57	0.007
Ln PVC	0.84	0.70–0.99	0.04
Male	10.74	3.20–36.07	<0.001
Smoking	6.89	1.47–32.38	0.01
RV enlargement	6.59	2.55–17.08	<0.001
PAH	2.57	1.01–6.52	0.047
SDNN/10	0.72	0.60–0.87	0.001
SDANN/10	0.73	0.61–0.88	0.001
**Multivariate analysis**			
SDNN/10	0.59	0.45–0.78	<0.001
Male	17.72	3.49–89.90	0.001
TWI-P	3.76	1.04–13.58	0.04
TWI-I	4.77	1.07–21.18	0.04

ACM, arrhythmogenic cardiomyopathy; CI, confidence interval; PAH, pulmonary artery hypertension; PVC, premature ventricular complex; RV, right ventricular; SDANN, standard deviation of the average NN intervals; SDNN, standard deviation of all NN intervals; TWI-I, T wave inversion in inferior leads; TWI-P, T wave inversion in precordial leads; VT, ventricular tachycardia.

### Clinical value of standard deviation of all normal-normal intervals in predicting sustained ventricular tachycardia in arrhythmogenic cardiomyopathy patients

**FIGURE 3:**
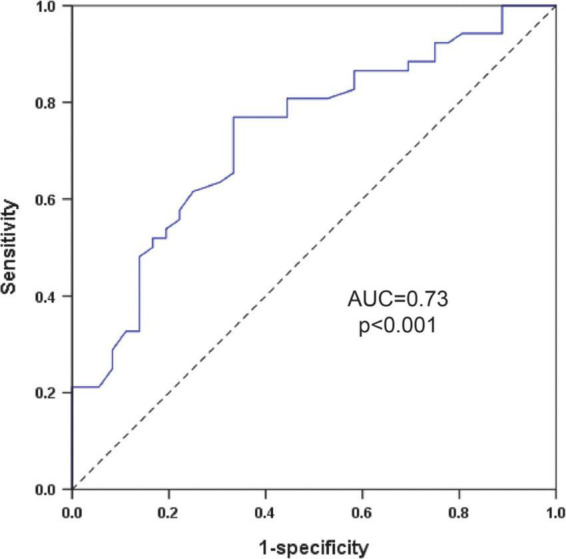
ROC curve to investigate the value of the SDNN in predicting sVT in patients with ACM. ACM, arrhythmogenic cardiomyopathy; AUC, the area under the curve; ROC, receiver operating characteristics; SDNN, standard deviation of all NN intervals; sVT, sustained ventricular tachycardia.

ROC curve was performed to value the level of SDNN in predicting sVT in patients with ACM. ROC curve showed the level of SDNN could significantly distinguish patients with sVT from patients without sVT [the area under the curve (AUC) = 0.73, 95% CI (0.63–0.84), *p* < 0.001, Figure 3]. The cutoff value of the SDNN level for predicting sVT in ACM patients was 126.5 ms based on the optimal balance between sensitivity and specificity. The level of SDNN < 126.5 ms predicted sVT with 76.9% sensitivity and 66.7% specificity. The positive and negative predictive value for sVT in ACM patients were also 76.9 and 66.7%, respectively.

### Validation of standard deviation of all normal-normal intervals in risk stratification for sustained ventricular tachycardia in arrhythmogenic cardiomyopathy patients

After excluding for variable reasons, 48 patients with ACM in the validation cohort were enrolled (Supplementary Figure 1). Patients in the validation cohort had a mean age of 52.4 years old and a high proportion of male (77.1%), and 30 patients (62.5%) experienced sVT. Patients with sVT had higher proportions of treatments with ICD (63.3% vs. 16.7%, *p* = 0.002), RFCA (50% vs. 11.1%, *p* = 0.006) and class III AADs (86.7% vs. 27.8%, *p* < 0.001). More detailed baseline characteristics of patients in validation cohort were shown in Supplementary Table 1.

**FIGURE 4:**
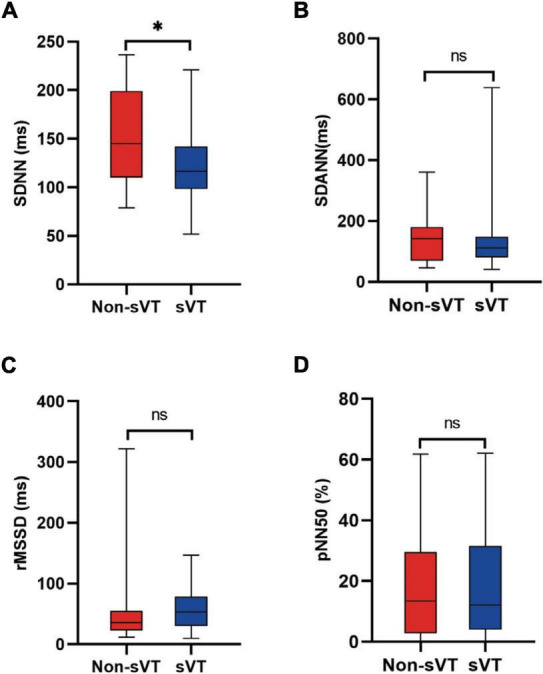
Comparison of HRV between ACM patients with and without sVT in the validation group. The Student’s *t*-test **(A)** and Mann-Whitney test **(B–D)** were used for assess the difference, *p < 0.05. ACM, arrhythmogenic cardiomyopathy; HRV, heart rate variability; pNN50, percentage of normal RR intervals that differ by > 50 ms; rMSSD, square root of the mean of the squares of the successive differences between adjacent NN intervals; SDANN, standard deviation of the average NN intervals; SDNN, standard deviation of all NN intervals; sVT, sustained ventricular tachycardia; ns, not significant.

Patients with sVT in the validation cohort had lower levels of SDNN (121.9 ± 39.2 vs. 154.3 ± 48.9 ms, *p* = 0.015), but similar levels of SDANN, rMSSD, and pNN50 (Figure 4). ROC curve showed the level of SDNN could also distinguish patients with sVT from patients without sVT [AUC = 0.69, 95% CI (0.53–0.89), *p* = 0.03, Supplementary Figure 3]. The level of SDNN < 126.5 ms predicted sVT with 66.7% sensitivity and 66.7% specificity in the validation cohort. The positive and negative predictive value for sVT in ACM patients were 76.9 and 54.5%, respectively.

## Discussion

The principal finding of our study is that decreased level of SDNN is not only in patients with ACM, but also is an independent predictor for sVT in patients with ACM. The key findings in our study were as follow: (1) Patients with ACM had impaired HRV, with decreased levels of SDNN, SDANN, rMSSD, and pNN50; (2) ACM patients with sVT had lower levels of SDNN, but similar levels of rMSSD and pNN50 compared with ACM patients without sVT; (3) SDNN was independently associated with sVT in patients with ACM, and could be used as a predictor of arrhythmogenic risk of ACM.

Cardiac sympathetic nerve fibers mainly stem from major autonomic ganglia along the cervical and thoracic spinal cord, and terminate on the surface of the heart to interact with myocardium (16). Cardiomyocyte necrosis and infiltration of inflammatory cells in the heart are expected to induce the necrosis of sympathetic nerve fibers and subsequent heterogeneous reinnervation, which might contribute to the overactivation of SNS in patients with ACM (4). In addition, the impaired hemodynamics due to frequent PVCs and scar formation in the right ventricular might also induce the activation of cardiac SNS along with withdrawal of parasympathetic tone in patients with ACM (17). Previous studies showed regional abnormal sympathetic innervation in ventricle and reduced myocardial adrenergic β receptors density in patients with ACM by means of SPECT/PET (9, 18). The present study employed HRV to assess the activity of cardiac SNS, and the results showed that patients with ACM had decreased levels of SDNN, SDANN, rMSSD, and pNN50 compared with healthy participants. All these results demonstrated that patients with ACM had excessive activation of cardiac SNS and impaired parasympathetic tone.

Another important finding of this study was that the level of SDNN was independently associated with sVT in patients with ACM, which indicated that overactivation of cardiac SNS might implicate in the process of ventricular arrhythmogenesis in ACM patients. There was evidence of abnormal intracellular calcium cycling in cardiomyocytes from ACM animals (19, 20), which might be accelerated by the overactivation of cardiac SNS (21). The stimulation of myocardial adrenergic β1 receptor could increase cellular Ca^2+^ loading through cyclic adenosine monophosphate (cAMP)—protein kinase A (PKA) mediated increasing of the L-type Ca^2+^ current, as well as SERCA mediated reducing of Ca^2+^ re-uptake. The resulting elevated diastolic Ca^2+^ levels in the cardiomyocyte may increase the probability of spontaneous sarcoplasmic reticulum (SR) Ca^2+^ release, followed by inducing of DAD and extra stimulus, which may be as triggers to induce sVT (22).

Replacement of injured cardiomyocytes with fibrofatty tissue in the ventricular walls is the distinct characteristic of ACM, and provide suitable substrate for sVT. The overactivation of cardiac SNS might accelerate the cardiomyocyte injury and subsequent fibrosis and scar formation (23). Adrenergic β1 receptors were shown to be localized within the intercalated disc in hearts from mice, and played positive adhesiotropy effects to maintain the integrity of cardiomyocytes under the circumstance of cardiac SNS activation (24, 25). However, the stimulation of adrenergic β1 receptor within the intercalated disc may accelerate the desmosome dysfunction in patients with ACM, which leads to cardiomyocyte injury, subsequent fibrosis and scar formation. In addition, the heterogeneous sympathetic never reinnervation of cardiomyocytes might promote the dispersion of APD, which provide suitable substrate for maintaining of sVT (26). Therefore, the overaction of cardiac SNS not only provides triggers, but also suitable substrate for the sVT in patients with ACM.

In addition, this study also identified the level of SDNN as a predictor of sVT in patients with ACM. Identification of patients with high risk of SCD is the primary goal in management of ACM patients. During the past decades, some predictors based on demography, genetics, surface ECG and echocardiography have been identified to screen ACM patients with high risk of SCD (3, 27–29). This study identified SDNN as a new predictor of sVT in ACM patients. The ROC curve showed SDNN had clinical values in differentiating ACM patients with sVT from those without sVT, which was validated in another independent cohort with ACM patients. All these results indicated the feasibility and reliability of SDNN to risk-stratify in patients with ACM in clinical practice. In addition, it might be more convenient to utilize SDNN in risk stratification in ACM patients compared with other established predictors since it was easily to be obtained. Lastly, the predictive value of SDNN was based on the overactivation of cardiac SNS, which was different from previous predictors. The SDNN level might provide some additional value to the present predictors in identifying ACM patients with high risk of SCD.

For its considerable role in ACM, cardiac SNS should be considered as an important therapeutic target in patients of ACM. In addition to adrenergic β-receptor blockers, other non-pharmacological therapy targeting cardiac SNS should also be considered in the management of ACM patients with refractory VAs. One small size clinical study showed that bilateral cardiac sympathetic denervation had beneficial effects in ACM patients with refractory VT (30). Further studies are needed to investigate the benefits of non-pharmacological therapy in ACM patients with VAs. In addition, the traditional Chinese medicine, which has been used in patients with cardiac dysfunction and VAs (31), might have potential benefits in patients with ACM. Shensongyangxin capsule was shown to have beneficial effects on VAs in patients with or without structural heart disease, and on HRV in patients with heart failure (32, 33). Wenxin Keli is another kind of traditional Chinese medicine which has been approved for arrhythmias, and was shown to induce the downregulation of adrenergic receptor genes (34). Therefore, the traditional Chinese medicine might be new option in management of patients with ACM.

## Study limitation

There are several limitations of our study. Firstly, the frequency domain analyses for HRV were not used in this study, because only 40% ACM patients in this study had data of frequency domain analyses. Therefore, this study did not investigate value of frequency domain analyses in patients with ACM. Secondly, the relatively small size of study population might have influences on the validation of our study findings. In addition, this is a retrospective study and has some disadvantages compared with prospective studies. Therefore, further prospective studies are needed to validate the value of SDNN in risk stratification for VAs in patients with ACM.

## Conclusion

The present study suggests that HRV is impaired in patients with ACM, and the SDNN level has a moderate value in risk stratification for sVT in ACM patients. In addition, the finding might provide new target for the further management of ACM with integrated traditional Chinese and western medicine.

## Data availability statement

The raw data supporting the conclusions of this article will be made available by the authors, without undue reservation.

## Ethics statement

The studies involving human participants were reviewed and approved by the Institutional Ethics Committee Board of the First Affiliated Hospital of Nanjing Medical University. Written informed consent for participation was not required for this study in accordance with the national legislation and the institutional requirements.

## Author contributions

BZ, XL, JY, AT, and BY: conception and design. JL, JLZ, CJ, MT, and XZ: administrative support. JL, JLZ, CJ, MT, XZ, AT, and BY: provision of study patients. BZ, CZ, DC, JZ, ZC, and FF: collection and assembly of data. BZ, CZ, AT, and BY: data analysis and interpretation. All authors: manuscript writing and final approval of manuscript.
