# Supplementary material for: Impaired heart rate variability in patients with arrhythmogenic cardiomyopathy: A multicenter retrospective study in China
Source: Front Cardiovasc Med. 2022 Oct 31;9:1044797. doi: 10.3389/fcvm.2022.1044797 (PMC9659603; doi:10.3389/fcvm.2022.1044797)

**Table S1. Clinical characteristics of ACM patients with in the validation group**

**Fig. S1. Study flow diagram**

ACM, arrhythmogenic cardiomyopathy; LVEF, left ventricular ejection fraction; VAs, ventricular arrhythmias; sVT, sustained ventricular tachycardia.

**Fig. S2. Representative ECGs from healthy control participant (A) and ACM patient (B) to show NN interval**

ECG, electrocardiography; ACM, arrhythmogenic cardiomyopathy.

**Fig. S3. ROC curve to investigate the value of the SDNN in predicting sVT in the validation cohort.**

ACM, arrhythmogenic cardiomyopathy; AUC, the area under the curve; ROC, receiver operating characteristics; SDNN, standard deviation of all NN intervals; sVT, sustained ventricular tachycardia.

**Table S1. Clinical characteristics of ACM patients with in the validation group**

| Variables | Overall  (n = 48) | Sustained VT  (n = 30) | No sustained VT  (n = 18) | p value |
| --- | --- | --- | --- | --- |
| Age (years) | 52.4 ± 17.3 | 52.7 ± 17.5 | 51.9 ± 17.3 | 0.89 |
| Male (n, %) | 37 (77.1) | 24 (80) | 13 (72.2) | 0.72 |
| History (years) | 7.5 (3.0, 14.0) | 8.5 (3.8, 14.0) | 7.0 (1.0, 13.0) | 0.44 |
| Age of diagnosis (years) | 43.7 ± 15.8 | 43.7 ± 15.7 | 43.8 ± 16.5 | 0.97 |
| Syncope (n, %) | 22 (45.8) | 13 (43.3) | 9 (50) | 0.65 |
| Family history (n, %) | 1 (2.1) | 0 (0) | 1 (5.6) | 0.38 |
| ICD (n, %) | 22 (45.8) | 19 (63.3) | 3 (16.7) | 0.002 |
| RFCA (n, %) | 17 (35.4) | 15 (50) | 2 (11.1) | 0.006 |
| Hypertension (n, %) | 8 (16.7) | 5 (16.7) | 3 (16.7) | 1.0 |
| Diabetes (n, %) | 0 (0) | 0 (0) | 0 (0) | NA |
| CAD (n, %) | 0 (0) | 0 (0) | 0 (0) | NA |
| Heart failure (n, %) | 8 (16.7) | 3 (10) | 5 (27.8) | 0.13 |
| Smoking (n, %) | 11 (22.9) | 9 (30) | 2 (11.1) | 0.17 |
| Alcohol (n, %) | 5 (10.4) | 5 (16.7) | 0 (0) | 0.14 |
| **Treatment with AADs** | | | | |
| Class I AADs (n, %) | 3 (6.3) | 0 (0) | 3 (16.7) | 0.047 |
| Class Ⅱ AADs (n, %) | 36 (75) | 25 (83.3) | 11 (61.1) | 0.10 |
| Class Ⅲ AADs (n, %) | 31 (64.6) | 26 (86.7) | 5 (27.8) | < 0.001 |
| Class Ⅳ AADs (n, %) | 1 (2.1) | 1 (3.1) | 0 (0) | 1.0 |
| **Surface electrocardiography** | | | | |
| TWI-P (n, %) | 33 (68.8) | 22 (73.3) | 11 (61.1) | 0.38 |
| TWI-I (n, %) | 24 (50) | 13 (43.3) | 11 (61.1) | 0.37 |
| RBBB (n, %) | 24 (50) | 15 (50) | 9 (50) | 1.0 |
| **Measurements on Echocardiography** | | | | |
| LVDD (mm) | 47.0 (40.0, 49.8) | 44.5 (38.0, 48.0) | 48.3 (43.8, 51.0) | 0.02 |
| LVEF (%) | 53.5 ± 6.1 | 53.9 ± 5.7 | 52.9 ± 6.8 | 0.62 |
| RV enlargement (n, %) | 19 (36.6) | 15 (50) | 4 (22.2) | 0.057 |
| RVOT dyskinesia (n, %) | 27 (56.3) | 18 (60) | 9 (50) | 0.50 |
| RV free wall dyskinesia (n, %) | 30 (62.5) | 22 (73.3) | 8 (44.4) | 0.045 |
| PAH (n, %) | 10 (20.8) | 6 (20) | 4 (22.2) | 1.0 |
| TR (n, %) | 32 (66.7) | 21 (70) | 11 (61.1) | 0.53 |
| MR (n, %) | 14 (28.6) | 9 (30) | 5 (26.3) | 0.78 |
| **Measurements on Holter** | | | | |
| PVCs (24h) | 616 (108, 4903) | 577 (93, 4519) | 720 (81, 11539) | 0.75 |
| NSVT (n, %) | 41 (46.6) | 24 (46.2) | 17 (47.2) | 0.92 |

AAD, anti-arrhythmic drug; ACM, arrhythmogenic cardiomyopathy; bpm, beats per minute; CAD, coronary artery disease; HR, heart rate; ICD, implanted cardiac defibrillator; LVDD, left ventricular end diastolic dimension; LVEF, left ventricular ejection fraction; MR, mitral regurgitation; NSVT, non-sustained ventricular tachycardia; PAH, pulmonary artery hypertension; PVC, premature ventricular complex; RBBB, right bundle branch block; RFCA, radiofrequency catheter ablation; RV, right ventricular; RVOT, right ventricular outflow tract; TR, tricuspid regurgitation; TWI-I, T wave inversion in inferior leads; TWI-P, T wave inversion in precordial leads; VT, ventricular tachycardia.

Fig. S1


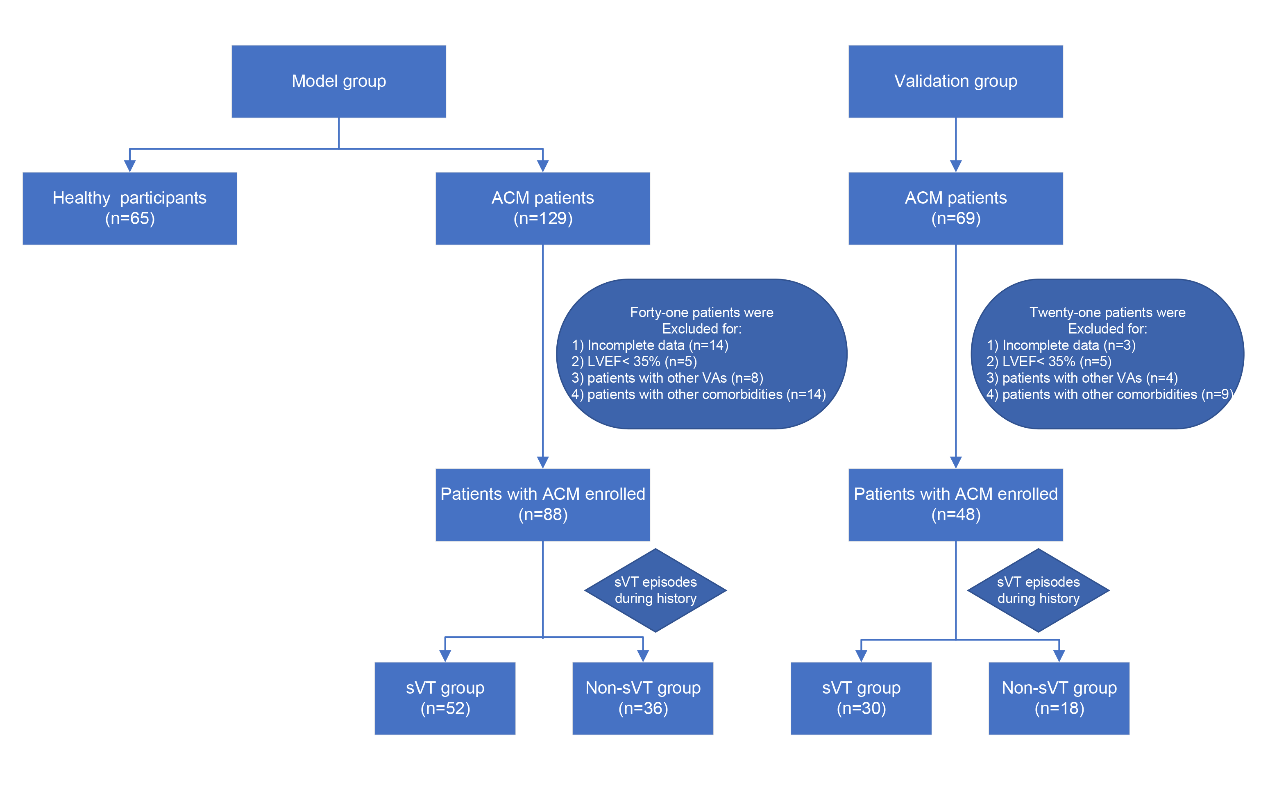


Fig. S2


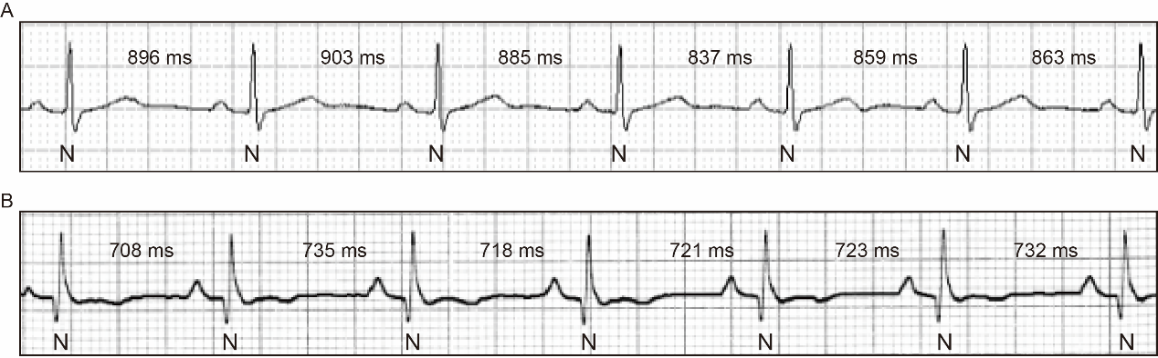


Figure S3


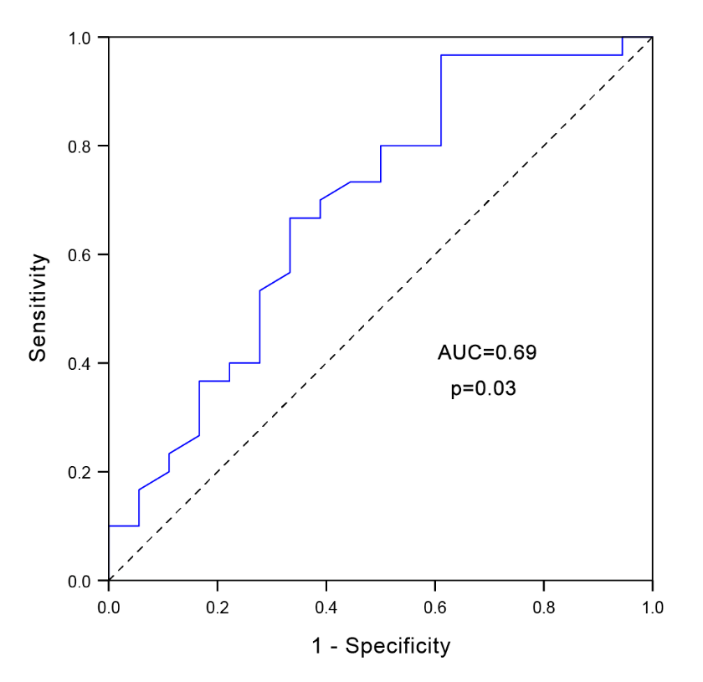

Supplement: Supplementary file 1 [file Data_Sheet_1.docx]
